# Spontaneous globe subluxation: a case report and review of the literature

**DOI:** 10.1186/s12245-021-00398-x

**Published:** 2021-12-18

**Authors:** Tesfaye Yadete, Ian Isby, Ketan Patel, Alex Lin

**Affiliations:** 1 Kirk Kerkorian School of Medicine at UNLV, 2040 W Charleston Blvd 3rd Floor, Las Vegas, NV 89102 USA; 2 Department of Emergency Medicine, Kirk Kerkorian School of Medicine at UNLV, 901 Rancho Lane, Ste 135, Las Vegas, NV 89106 USA

**Keywords:** Spontaneous globe subluxation, Graves’ orbitopathy, Idiopathic Intracranial Hypertension, floppy eyelid syndrome, globe reduction, Hypertension

## Abstract

**Background:**

Spontaneous globe subluxation (SGS) is an atraumatic anterior dislocation of the eyeball. It is exceedingly rare. Understanding SGS predisposing factors may help uncover its etiology and undertake vision-saving management.

**Case presentation:**

A 48-year-old female presented to the ED with her right eye out of its socket. She reported blurry vision, photophobia, and pain in the affected eye. She was unable to close her right eyelid and was in obvious distress. On arrival, her blood pressure was elevated. Her medical history was notable for hypertension and obesity. On physical examination, extraocular eye movements were not intact, and the globe appeared whole and round. She was also unable to count fingers with the affected eye. There was no visible trauma to the face. Multiple wet gauzes with sterile saline were placed over the displaced eyeball. Direct and even pressure was applied on the globe. Within 30 s, the globe was reduced back in. The patient was able to close her eyelids and reports substantial pain relief with reduction. A CT scan of the orbits was then obtained, demonstrating mild bilateral proptosis. The globes were normal and symmetric. No intraconal or extraconal abscess or infection was seen. There were no intraconal or extraconal masses. There was no acute orbital traumatic injury, no avulsion of the optic nerve, ocular rupture, or retrobulbar hematoma. After reviewing the case with an ophthalmologist, a follow-up appointment with the ophthalmologist was arranged. The patient was discharged on erythromycin ointment. Post-discharge investigation of the CT imaging revealed dilated optic nerve sheaths, tortuosity of the optic nerve, and empty sella.

**Conclusions:**

In addition to causing distress and severe anxiety, SGS poses numerous immediate as well as long-term complications. Traction of the optic nerve and retinal vasculature may potentially cause retinal venous congestion and loss of visual acuity with potential vision loss. In the absence of known risk factors or disease processes, orbital imaging and serological studies for thyroid ophthalmopathy should be considered.

## Background

Spontaneous subluxation of the globe is an unsettling presentation not seen frequently in the emergency department (ED). Globe subluxation is classified as traumatic, spontaneous, or voluntary. It is a clinical diagnosis; however, imaging is often utilized in attempting to establish the cause. Reduction is typically done manually and less frequently requires surgical management. Ocular examination, reduction techniques, and post-reduction management are well within the scope of practice for emergency physicians, and these techniques can be safely performed in the emergency department.

## Case report

A 48-year-old female presented to the emergency department (ED) shortly after her right “eyeball popped out” of its socket while rubbing her eye. The patient endorsed blurry vision, photophobia, and pain in the affected eye. She was unable to close her right eyelid and was in obvious distress. The patient had no history of globe subluxation, no recent trauma, and did not use contact lenses or wear corrective lenses. On arrival, her vital signs were notable for a blood pressure of 180/110. This improved to 152/101 during her ED stay without medical intervention. All other vital signs were within normal range. Her medical history was notable for hypertension and obesity, with a BMI of 45 kg/m^2^. She endorsed a history of smokeless tobacco, alcohol, and drug use.

**Fig. 1:**
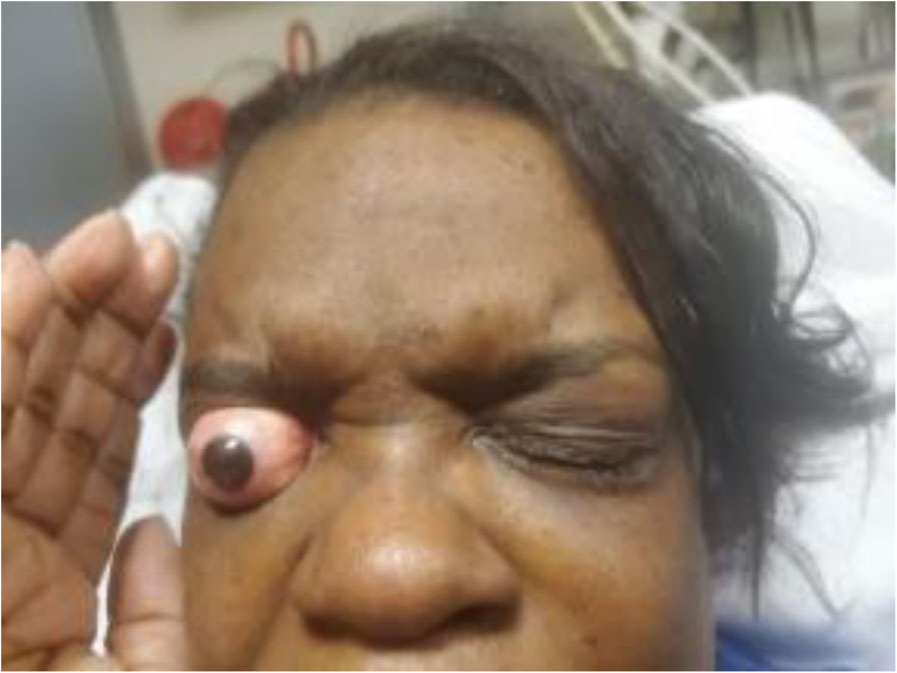
Patient with right eye globe subluxation

On physical examination, the right eye was subluxed anteriorly and sitting outside the orbit (see Fig. 1). Extraocular eye movements were not intact, and the globe appeared whole and round. On visual acuity exam, she was unable to count fingers with the affected eye. The examination of her left eye was grossly normal. There was no visible trauma to the face.

Following a brief evaluation, multiple wet 4 × 4 gauzes with sterile saline were placed over the displaced eyeball. The patient was instructed to look down while applying direct and even pressure. Within 30 s, the globe was reduced into the orbit. Significant subconjunctival edema was observed on post-reduction examination; however, she was able to close her eyelids. She reported feeling pressure during reduction, followed by substantial pain relief with reduction. The patient was advised to place her hand over her right eye and maintain constant light pressure.

**Fig. 2:**
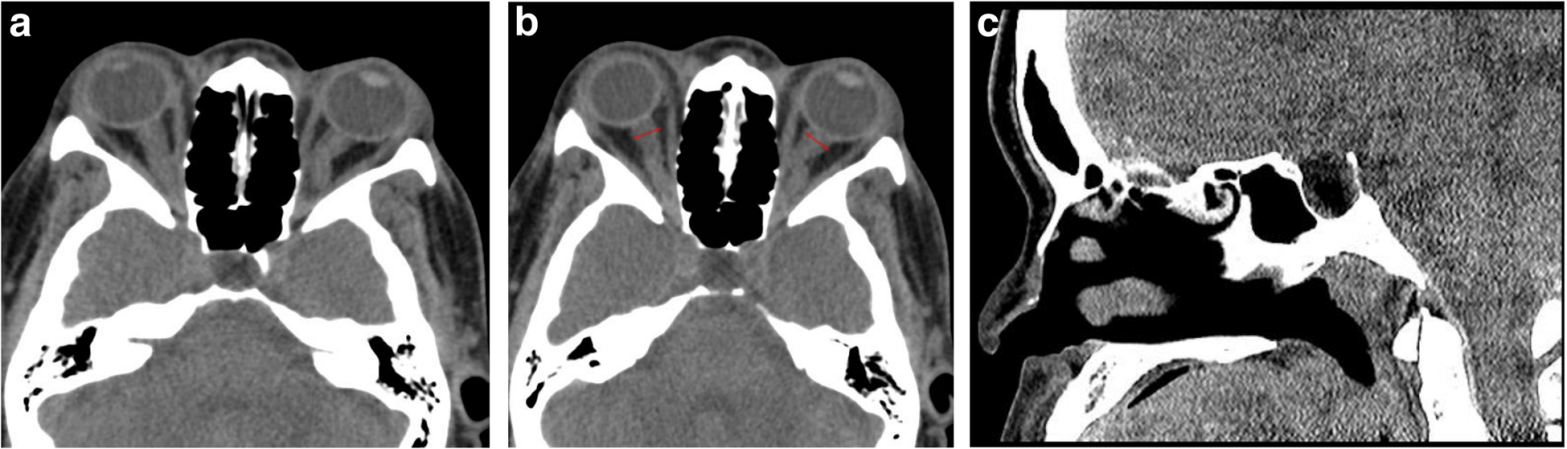
**a** Bilateral proptosis and kinking or tortuosity of the optic nerve. **b** Significantly dilated optic nerve sheaths. **c** Empty sella

A non-contrast computed tomography (CT) scan of the orbits was subsequently obtained, demonstrating mild bilateral proptosis (see Fig. 2). The extraocular muscles, optic nerves, and chiasm were normal and symmetric. The globes were normal and symmetric. No intraconal or extraconal abscess or infection was seen. There were no intraconal or extraconal lesions or masses. The visualized intracranial contents were without abnormality. There was no acute orbital traumatic injury, no avulsion of the optic nerve, ocular rupture, or retrobulbar hematoma. No orbital fracture was seen. Fluorescein staining and slit lamp examination was performed and showed a corneal abrasion. After discussing the case with an ophthalmologist, the patient was discharged on erythromycin ointment. A close follow-up appointment with the ophthalmologist was arranged, and the patient was asked to follow-up with her primary care physician. Post-discharge investigation of the CT imaging revealed dilated optic nerve sheaths, tortuosity of the optic nerve, and empty sella.

## Discussion and conclusion

The luxation of a globe results when the globe’s equator bulges out anteriorly past the eyelid aperture. The contraction of the orbicularis muscle further displaces the globe anteriorly, causing it to be trapped outside the eyelid aperture, which further limits spontaneous reduction and extraocular movement [1–4]. The most common risk factor associated with spontaneous globe luxation (SGL) is proptosis from having shallow orbits or space-invading retrobulbar lesions [3]. Other reported predisposing factors linked with SGL are structural anomalies such as a relaxed supporting fascia, orbital septum, or laxed extraocular muscles [5, 6]. In one study, malar hypoplasia is reported as the most common predisposing factor for SGL [7]. In malar hypoplasia, the apex of the cornea is often present anterior to the malar prominence predisposing anterior globe luxation. In 2002, Kunesh and Katz described exophthalmos secondary to Graves’ orbitopathy as the most common predisposing factor in globe luxation [1]. Though infrequently reported, exophthalmos secondary to orbital fat hypertrophy is also noted as a predisposing factor in obese patients [7].

SGL can be caused by non-traumatic eye manipulations, as in the case presented. It can also be caused by forceful Valsalva or eyelid maneuvering during contact lens insertion, as users push the eyelids past their natural threshold [1, 3]. In the case of voluntary globe luxation (VGL), participants initially experience SGL and later learn to sublux their globes without manipulation just by using their extraocular muscles [8, 9]. A small number of cases have also been reported of self-enucleation of the globe by “gouging” in patients with mental illness [10–13]. Though rare, traumatic globe subluxation (TGS) occurs secondary to significant traumatic events that damage the orbit and push the globe anteriorly. In the ED, TGS may present following high energy motor vehicle accidents and can also be seen when someone grips the eyeballs during a fight or secondary to birth trauma with compression of the cranium [14–22].

The pathogenesis of SGL is not fully elucidated. However, different pathological processes and risk factors have been reported. Graves' ophthalmopathy is the most common cause of space-occupying disease leading to SGL. SGL has been linked with floppy eyelid syndrome (FES) [2, 5, 13]. FES is described as saggy eyelids accompanying punctate epithelial keratopathy, ptosis of lateral eyelashes, and characteristic conjunctival changes. Hashimoto’s disease [2] and hyperemesis gravidarum, which causes shallow ocular space secondary to extraocular muscles hemorrhage and Valsalva pressure, are also linked to causing SGL [3]. Other disease processes implicated to cause SGL include arteriovenous malformations, Engelmann’s disease, histiocytosis X, orbital tumors, and unusually large globes [3, 23, 24]. In 2012, Kumar et al. reported the first case of SGL associated with chronic obstructive pulmonary disease [6]. They hypothesized that the raised intrathoracic pressure and a subsequent increase in intraorbital pressure might have instigated the luxation. In 2015, Ortega-Evangelio et al. reported SGL secondary to iatrogenic Cushing syndrome [25]. A case of globe luxation was also reported on a patient under otherwise uneventful general anesthesia [26].

There is a higher frequency of globe luxation in African American individuals due to a higher likelihood of these populations having shallow orbits [3, 26]. Obesity is an important risk factor that causes exophthalmos and is also associated with FES in patients [7, 13, 26]. However, other reports have also shown that FES can occur in individuals with normal BMI [27]. In the case presented, the patient was African American and obese. Additionally, the patient had a history of uncontrolled hypertension, another risk factor for FES [27]. Some etiologies were more likely than others for this patient. It was hypothesized that the patient might have undiagnosed Grave’s disease given the bilateral proptosis observed on exam and CT. A revisiting of the CT imaging with a radiologist demonstrated dilated optic nerve sheaths, tortuosity of the optic nerve, and empty sella, which are the most common imaging findings in a patient with idiopathic intracranial hypertension [28, 29]. In addition to these imaging findings, the patient’s body habitus, female gender, and presented visual disturbance make for a high differential diagnosis of mild IIH. As far as we know, there has not been a reported case of IIH-associated SGL. Other etiologies as a cause for SGL in our patient include undiagnosed FES, exophthalmos in the context of fat hypertrophy, or another undiagnosed medical condition that could have predisposed her to SGL.

In addition to causing distress and severe anxiety, SGL poses numerous immediate as well as long-term complications. In this case, the patient had reported pain, photophobia, and blurry vision. Globe exposure may result in keratitis and blepharospasm. Traction of the optic nerve and retinal vasculature may potentially cause retinal venous congestion and loss of visual acuity with potential vision loss [1, 3, 9, 13]. Hence, timely reduction of the globe may help reduce the likelihood of optic nerve complications. There has been a case report of bilateral optic neuropathy linked with floppy eye syndrome and voluntary globe luxation [13]. An additional report discusses SGL-induced optic neuropathy as a subsequent complication as well [30].

Uncomplicated globe subluxation can be reduced relatively easily. Before any attempt to reduce the globe, an ocular exam including acuity, pupillary reflex, and extraocular movement at minimum is warranted. A successful globe reduction can be facilitated by encouraging the patient to relax. The use of anxiolytics, analgesics, and topical ocular anesthetic agents further eases the reduction process [3]. In the case presented, no anxiolytic, analgesic, or anesthetic agents were used before reduction, which could have helped the patient’s level of anxiety and pain. If the eyelids are retracted behind the displaced globe, advise the patient to look down before retracting the eyelids. Simultaneously, apply moderate and continuous pressure posteriorly and downward direction of the sclera [26, 31]. In this case, the patient’s eyelid was not retracted behind the subluxated globe, making the reduction easier. It is crucial to consider infiltrative diseases and orbital tumors in patients with SGL. If an attempt to reduce the globe fails, urgent ophthalmologist consultation is indicated.

Globe luxation can also be managed surgically by performing lateral tarsorrhaphy. However, this procedure has been reported to predispose subsequent luxation because it creates a tighter orbit and elevated intraorbital pressure. Furthermore, if subluxation reoccurs, the reduced palpebral aperture will make performing another reduction very challenging [24]. An alternative surgical procedure is orbital decompression. Lumbreras-Fernández et al. reported the superiority of orbital decompression to tarsorrhaphy in managing patients with SGL and exophthalmos [7]. Luckily, most cases of SGL require no surgical intervention.

Following the reduction of the globe, treating the underlying may help prevent future subluxation [3, 7, 25]. If no reported risk factors or identifiable triggering medical or psychiatric illnesses present, orbital imaging or serological studies for thyroid ophthalmopathy should be considered. Post-reduction outpatient follow-up with an ophthalmologist and primary care provider can aid uncover the underlying etiology and predisposing factors.

Globe subluxation generates anxiety, both to the patients and to unfamiliar providers in the ED because many ED nurses and doctors will not have treated it before. Globe subluxation is diagnosed clinically as the luxation is observable to the naked eye. Safeguarding visual function should be the primary target, and immediate diagnosis and action are essential to protecting patient vision. Hence, providers must familiarize themselves with the risk factors and associated disease processes. Knowledge of pre-reduction eye examination, globe reduction procedure, and post-reduction management are critical elements in SGL treatment and prevention of reoccurrence.

## Data Availability

Not applicable

## Abbreviations

SGS: Spontaneous globe subluxation
VGL: Voluntary globe luxation
TGL: Traumatic globe luxation
ED: Emergency department
SGL: Spontaneous globe luxation
FES: Floppy eyelid syndrome
CT: Computed tomography
